# Massive Sequencing of V3-V4 Hypervariable Region in Pyogenic Liver Abscesses Reveals the Presence of Unusual Bacteria Not Detected by Classical Culture Methods

**DOI:** 10.3390/microorganisms13010131

**Published:** 2025-01-10

**Authors:** Verónica Fernández-Sánchez, Estibeyesbo Said Plascencia-Nieto, Mónica Alethia Cureño-Díaz, Emilio Mariano Durán-Manuel, Aida Verónica Rodríguez-Tovar, Claudia Camelia Calzada-Mendoza, Clemente Cruz-Cruz, Miguel Ángel Loyola-Cruz, María Elizbeth Álvarez-Sánchez, Juan Carlos Bravata-Alcántara, Enzo Vásquez-Jiménez, Víctor Hugo Gutiérrez-Muñoz, Dulce Milagros Razo Blanco-Hernández, Liliana Nicolás-Sayago, Araceli Rojas-Bernabé, Omar García-Hernández, Erika Gómez-Zamora, Mireya Ruíz-Valdés, Graciela Castro-Escarpulli, Juan Manuel Bello-López

**Affiliations:** 1 Hospital Juárez de México, Mexico City 07760, Mexico; 2 Facultad de Estudios Superiores Iztacala, Universidad Nacional Autónoma de México, Mexico City 54090, Mexico; 3 Sección de Estudios de Posgrado e Investigación, Escuela Superior de Medicina, Instituto Politécnico Nacional, Mexico City 11340, Mexico; 4 Escuela Nacional de Ciencias Biológicas, Instituto Politécnico Nacional, Mexico City 11340, Mexico; 5 Laboratorio de Patogénesis Celular y Molecular Humana y Veterinaria, Posgrado en Ciencias Genómicas, Universidad Autónoma de la Ciudad de México, Mexico City 03100, Mexico; 6 División de Investigación, Facultad de Medicina, Universidad Nacional Autónoma de México, Mexico City 54090, Mexico

**Keywords:** bioinformatics, DNA sequencing, V3-V4 hypervariable, pyogenic liver abscesses

## Abstract

Pyogenic liver abscesses (PLAs) are serious infections in which doctors often fail in identifying the causative agent due to microbiological limitations. These limitations in detecting uncommon pathogens complicate the treatment and recovery. Molecular techniques, like massive sequencing, enable the detection of uncommon pathogens and highlight the shortcomings of traditional cultures. The aim of this work was to characterise the bacterial composition of PLAs through massive sequencing of the V3-V4 hypervariable region of the *16S rRNA* gene in cases where conventional culture methods were negative. Purulent material was collected from three patients with PLAs at Hospital Juárez de México. Classical and molecular microbiological cultures were performed in parallel. Metagenomic DNA was extracted and massively sequenced (*16S rRNA* gene) using the Illumina MiSeq platform. A bioinformatic analysis was performed to determine the diversity at six different taxa levels and the relative abundances. The culture methods were not sufficient to detect the causative agent of the PLAs. However, the massive sequencing revealed the causative agents of the monomicrobial and polymicrobial infectious foci, with *Gardnerella vaginalis*, *Lactobacillus iners*, and *Prevotella timonensis* as the dominant bacteria. The massive sequencing revealed the presence of unusual pathogens that traditional culture failed to detect. There is an immediate need for molecular or comprehensive microbiological culture techniques to search for unusual bacteria in the diagnosis of PLAs.

## 1. Introduction

Hepatic abscesses (HAs) are suppurative infections caused by the invasion and multiplication of microorganisms within the liver parenchyma [1]. Although they are uncommon in the general population, in developing countries, their incidence is higher, due to factors such as poor sanitation, poor access to water, poor health services, the presence of comorbidities, and inaccurate diagnoses of HAs, among others [2,3,4]. Hepatic abscesses are mainly divided into three types according to the infectious aetiology: amoebic liver abscesses (ALAs), whose agent is the protozoan *Entamoeba histolytica* [5]; pyogenic liver abscesses (PLAs), caused by commensal bacteria, enterobacteria, and strict anaerobic bacteria [3,6,7]; and finally, fungal liver abscesses (FLAs), which have been associated with yeast-like fungi of the genera *Candida* spp. and *Cryptococcus* spp., and filamentous fungi of the genus *Aspergillus* spp. [8,9,10,11,12]. Hepatic abscesses are prevalent in regions where amoebiasis is endemic, and in countries such as Mexico, it remains one of the notable infectious diseases [2].

Epidemiological studies on the three types of HAs are scarce, and the few that exist indicate that the rates of PLAs are 4.7 and 2.7 cases per 100,000 inhabitants/year, for men and women, respectively, and that they increase significantly with age, especially between 50 and 64 years old, predominantly in men [13]. Other research indicated that men over 60 years of age are susceptible, and comorbidities serve as triggers [14]. Pyogenic liver abscesses are particularly dangerous in immunocompromised patients and patients with diabetes, malignant diseases, or those who have received a liver transplantation, as their health status makes them more susceptible to infections of this type [13,15]. Advances in imaging, interventional procedures, and antimicrobial treatment have significantly improved the outcomes in this condition, reducing mortality [16,17]. Nevertheless, an accurate diagnosis of PLAs remains a challenge due to the limitations of traditional culture methods as, in addition to their low sensitivity, these methods are performed in a targeted or purposive manner based on the most documented methods in the scientific literature. This is why it has been reported that in 30–38% of cases, culture methods fail to identify the pathogen [18,19]. The factors leading to false-negative results in the identification of the causative agent of PLAs include the following: early initiation of empirical antimicrobial therapy that may inhibit bacterial growth, polymicrobial infections, and insufficient culture methods, among others [19]. Among the most common bacterial pathogens of PLAs are enterobacteria, such as *Escherichia coli* and *Klebsiella* spp., anaerobes such as *Bacteroides* and *Clostridium* spp., and some genera of Gram-positive cocci [3,6,7].

Nonetheless, polymicrobial infections and false-negative cultures have driven the need for diagnostic techniques based on molecular methods. Polymerase chain reaction (PCR) has been one of the techniques used, as it allows for the direct detection of bacterial DNA in samples without the need for culturing. However, conventional end-point PCR in the study of PLAs has limitations, such as the need for pure cultures or specific primers [20,21,22,23]. This has an impact since, in PLAs with polymicrobial aetiology or false-negative cultures, PCR may not be sufficient to detect the bacterial diversity present. To address these limitations, massive *16S rRNA* gene sequencing has emerged as a powerful tool in the study of polymicrobial PLAs [24,25]. This metagenomic approach makes it possible to determine, through parameters of taxonomic diversity and relative abundance, the range of bacteria present in the infectious focus, regardless of their ability to grow in classical culture, and to detect uncommon bacteria that could be generating the infectious focus. This molecular technology has been shown to be powerful since the relative abundance measurements provided by this tool allows the predominance of the pathogen to be demonstrated, and the causative agent can be inferred based on this parameter. In contrast, massive sequencing successfully overcomes the limitations of previous attempts to determine bacterial diversity in PLAs using cloning and Sanger sequencing assays [26].

Therefore, the aim of the present study was to characterise cases of PLA by massive sequencing of the *16S rRNA* gene, where conventional culture methods were insufficient for the detection of the causative agent. The need for state-of-the-art molecular techniques or comprehensive microbiological culture techniques to search for uncommon bacteria in the diagnosis of PLA is analysed and discussed.

## 2. Materials and Methods

### 2.1. Origin of the PLA Patient Population

The Hospital Juárez de Mexico (HJM) is a third-level public health care institution with 54 medical specialties located in the north of Mexico City. The HJM serves an annual average of 30,000 patients with 391 census beds. We included three patients (two women and one man) who underwent drainage of HA of unknown aetiology in the General Surgery Department of the HJM during the period between December 2023 (*n* = 2) and January 2024 (*n* = 1). These patients presented symptomatically with features suggestive of HA (epigastric pain radiating to the hemicintestinal belt, abdominal pain in the right hypochondrium, chest pain radiating to the shoulder, vomiting, fever, anorexia, asthenia, adynamia, dyspnoea, and diaphoresis). Liver function tests, blood parameters, and coagulation times were altered. Patients with HA, confirmed by imaging (tomographic study of abdomen and pelvis), underwent open drainage, and a 20 mL sample of the purulent material was placed in a sterile plastic bottle and immediately transferred at 4 °C to the Bacteriology and Research laboratory for microbiological and molecular analyses, respectively. Bacteriological culture of the purulent material was performed within the first 2 h after its collection in the operating room.

**Figure 1:**
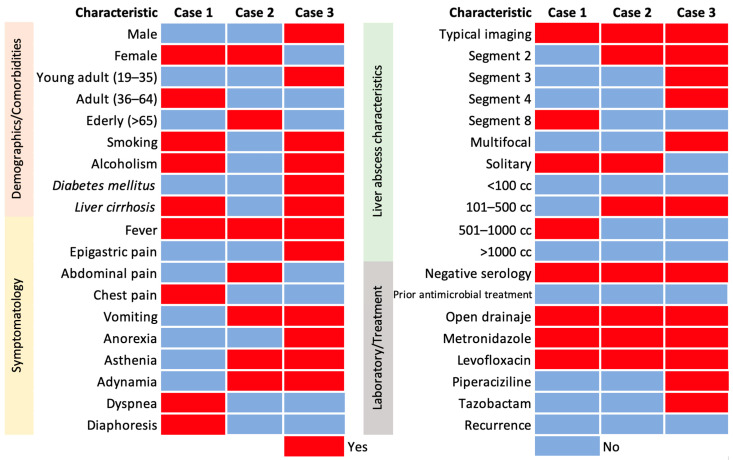
Demographic characteristics and comorbidities, symptomatology, HA characteristics, laboratory tests, and pharmacological treatment of patients with a diagnosis of HA seen at Hospital Juárez de México.

To rule out parasitic aetiology, the sera of the patients were tested for IgG antibodies using the commercial kit “*Entamoeba histolytica* IgG ELISA Test” (DGR international, Springfield, NJ, USA) according to the manufacturer’s instructions. Figure 1 shows the demographic characteristics and comorbidities, symptomatology, HA characteristics, laboratory tests, and pharmacological treatment of patients diagnosed with HA treated at HJM.

### 2.2. Metagenomic DNA Extraction and Quality Control

Two hundred microlitres of purulent material were subjected to extraction and purification of metagenomic DNA (per triplicate) using the DNeasy Blood and Tissue kit (Qiagen, Venlo, The Netherlands) with minor modifications. DNA integrity of metagenomic DNA was visualised on horizontal 0.8% agarose gels and the quality and concentration of DNA was evaluated according to absorbance ranges (260/280 nm). Replicates of metagenomic DNA were pooled (per sample) and were subjected to end-point PCR assays using 27F/1492R primers (V1-V9 regions) to determine if DNA samples were amplifiable (the full *16S rRNA*; 1492 bp amplicon) [27]. Finally, metagenomic DNA samples were subjected to massive sequencing using the Illumina platform, as follows.

### 2.3. Massive Metagenomic DNA Sequencing with the Illumina Platform

The hypervariable region V3-V4 of the *16S rRNA* gene was amplified by PCR using the primers 341F/805R previously reported by Klindworth et al. [28]. Library preparation was performed according to the Illumina 16S metagenomic sequencing protocol. Briefly, *16S rRNA* amplicons were purified with the DNA clean and concentrator kit (Zymo Research, Irvine, CA, USA). Dual indices and Illumina sequencing adapters were attached in a second PCR step using the Nextera XT Index kit V2 (Illumina, San Diego, CA, USA).

**Figure 2:**
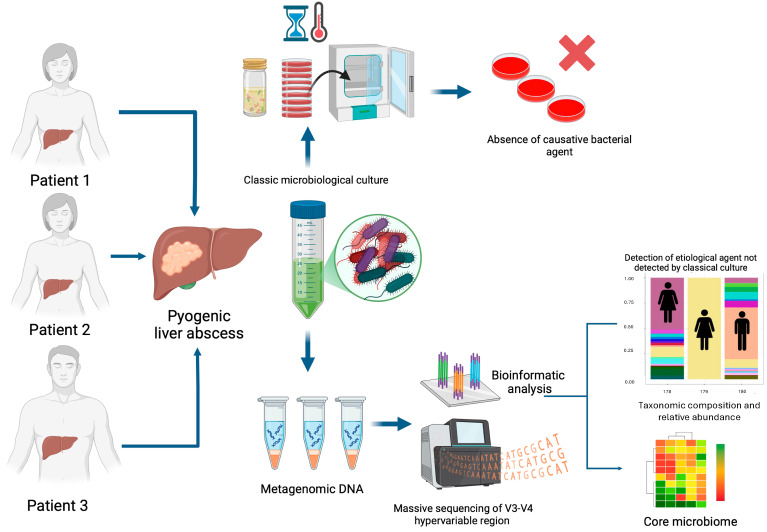
Workflow in the investigation of the aetiology of PLAs (microbiological culture and massive sequencing by Illumina platform) of patients with a diagnosis of HA seen at Hospital Juárez de México.

Finally, amplicons were purified using AMPure XP beads (Beckman Coulter, Brea, CA, USA), pooled in equimolar concentrations, and sequenced on a MiSeq Illumina instrument. The workflow of the investigation of the aetiology of PLAs (microbiological culture and massive sequencing by Illumina platform) is shown in Figure 2.

### 2.4. Analysis of PLA Sequence Data

#### 2.4.1. Quality Control of Sequencing Data

The DADA2 programme was used for the quality control review of PLA sequencing data to determine the overall status of information obtained and to take corresponding measures to filter the information [29]. For this purpose, a sequence-by-base analysis was performed by considering intervals of sequence quality values per base at each data position in FASTQ. Graphs of read length ratio and phred values were generated.

#### 2.4.2. Assignment of Taxonomic Parameters and Relative Abundance

Illumina raw sequences (38,542 sequences) were analysed under the DADA2 for filtering analysis, dereplication, chimaera identification, and paired-read joining. The database with the highest species-level annotation for each HA case dataset (SILVA nr99, version 138.1, with 510,984 total sequences) was used [30]. The generated input files were processed in the R package phyloseq for final taxonomic assignment at the different levels (phylum, class, order, family, genus, and species) [31].

#### 2.4.3. Analysis of the Core Microbiome in the PLA

To calculate the taxa present in the PLA cases so as to determine the predominant ones, an analysis was performed using the microbiome v1.17.3 package. The visualisation of the data was performed through heat maps with the plot core function. The variables used for the heat maps were the relative abundance and the output Amplicon Sequence Variants (ASVs) for the taxonomic groups studied (genus and species).

## 3. Results

### 3.1. Classical Microbiological Analysis of PLA

The classical microbiological culture analysis included direct seeding of purulent material on one set of culture media in aerobic conditions (blood agar, chocolate agar, MacConkey, salt and mannitol agar, and potato dextrose agar) and another set of the same media in anaerobic conditions. The results showed the absence of bacterial growth even though abundant Gram-negative short bacilli, Gram-variable, and Gram-positive long bacilli were observed under the microscope.

### 3.2. Quality Control of PLA Sequencing Data

**Figure 3:**
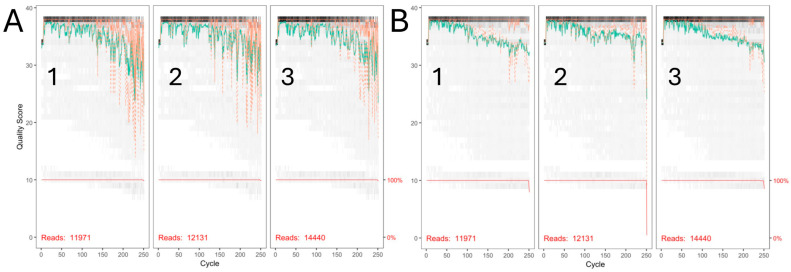
Analysis of sequence quality by base (Forward and Reverse) of the data obtained by massive sequencing of the *16s rRNA* gene of the PLAs of the patients treated at *Hospital Juárez de México*. (**A1**,**B1**): PLA S178 reads, (**A2**,**B2**): PLA S179, and (**A3**,**B3**): PLA S180. Green: High-quality sequencing reads; red: low-quality sequencing reads.

The DADA2 analysis of the quality control of the sequencing data obtained from the sequencing of the ribosomal libraries of the V3-V4 hypervariable region showed that most of the reads were of good quality above the phred quality score (score 30). This can be seen in Figure 3, which shows that the first 250 nucleotides that make up the reads (11,971, 12,131, and 14,440) are within the appropriate quality score.

### 3.3. Error Models in PLA Sequencing Data

**Figure 4:**
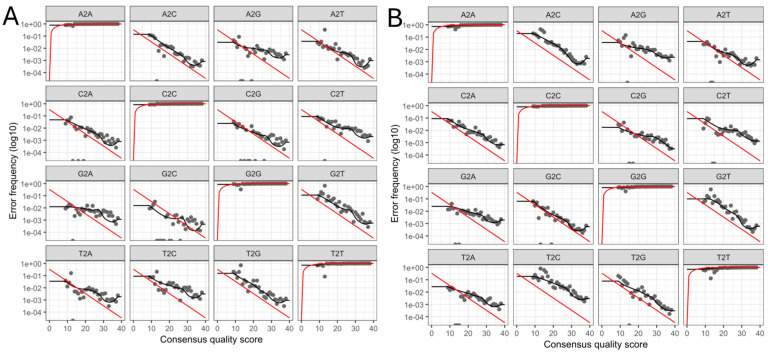
Analysis of the error rates for each possible nucleotide transition of the data obtained by massive sequencing of the 16s rRNA gene from the PLA of patients treated at Hospital Juárez de México. (**A**). Forward sequence and (**B**). Reverse sequence. Red line: Expected error rates under the nominal Q-score definition.

A parametric error model was run using the DADA2 algorithm to determine base transitions. Figure 4 shows the error rates for each possible nucleotide transition (A → C, A → G, etc.) where the observed (grey dots) and estimated (black lines) error rates after algorithm conversion are correctly matched. The red line shows the expected error rates under the nominal Q-score definition, where it is observed that they decrease as a function of higher quality, as expected. Therefore, the constructed error model is adequate, and we proceed to the analysis of taxonomic assignment and relative abundance.

### 3.4. Taxonomic Composition and Average Relative Abundance of PLAs

**Figure 5:**
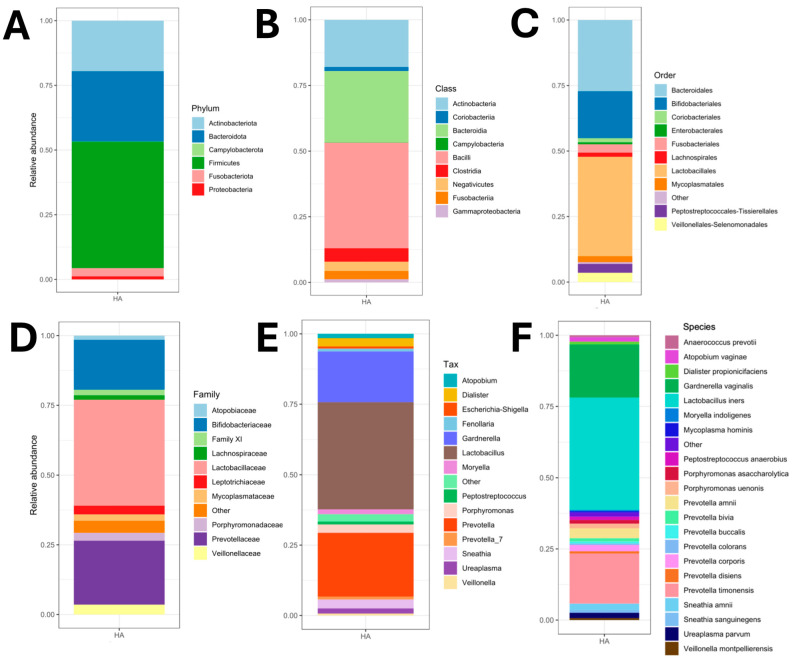
Average taxonomic composition of PLAs at six taxonomic levels and relative abundance of PLAs analysed in this study. (**A**) Phylum. (**B**) Class. (**C**) Order. (**D**) Family. (**E**) Genus. (**F**) Species level.

The average taxonomic composition of PLAs was reported at six taxonomic levels (from phylum to species). The results revealed that the PLAs consisted of six phyla, where Actinobacteriota, Bacteroidota, and Firmicutes were dominant, with relative abundances of ~0.23, ~0.30, and ~0.51, respectively. In contrast, for this population, the phyla Fusobacteria, Proteobacteria, and Campylobacterota showed the lowest relative abundances (Figure 5A). The predominant bacterial populations at the class level showed that within the phylum Actinobacteriota, the class Actinobacteria was dominant compared to the class Coriobacteriia. At the same taxonomic level, no class diversification was identified in the phylum Bacteroidota as only the class Bacteroidia was identified (Figure 5B). Finally, the predominant phylum (Firmicutes) was resolved into three classes (Bacilli, Clostridia, and Negativicutes), with the Bacilli class being the dominant one (Figure 5C). At the next taxonomic level, 11 orders were detected where Bacteroidales, Bifidobacteriales, and Lactobacillales were dominant (Figure 5D).

From the next taxon, three uncommon dominant families were detected as causative agents of PLA: *Bifidobacteriaceae* (~0.27), *Lactobacillaceae* (~0.52), and *Prevotellaceae* (~0.26). Lastly, the last two taxa that are related in the final genetic assignment and identity were bacteria that suggested a direct relationship with the sex of the patients (Figure 5E,F). The three predominant genera and species in the average analysis of taxonomic diversity and relative abundance showed *Gardnerella vaginalis* (~0.22), *Lactobacillus iners* (~0.51), and *Prevotella timonensis* (~0.24) as causative agents of PLA. Therefore, an analysis of taxonomic composition and relative abundance per patient was performed to confirm the infectious aetiology, as follows.

### 3.5. Taxonomic Composition and Relative Abundance by Individual PLA

**Figure 6:**
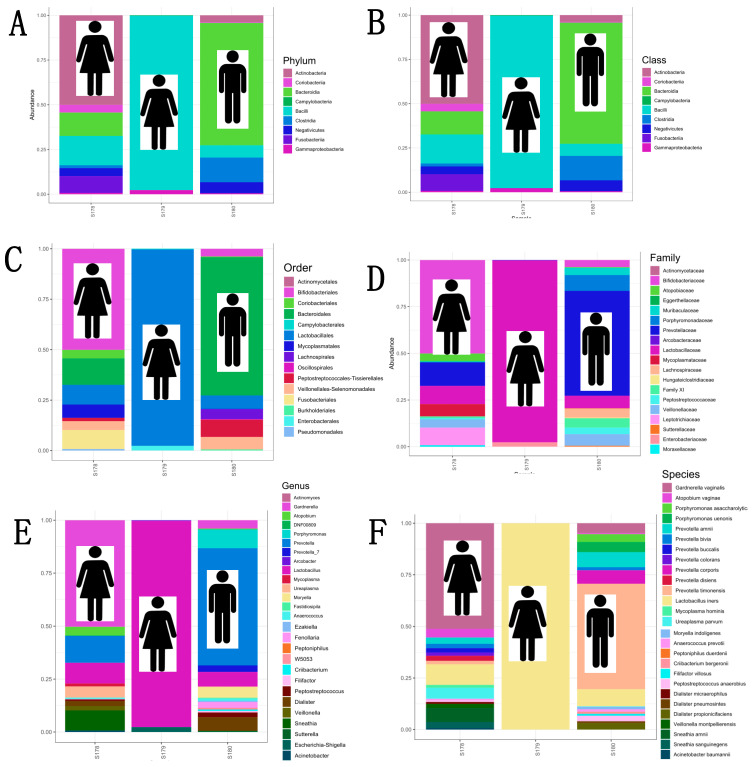
Individual taxonomic composition (by sex) of PLAs at six taxonomic levels and relative abundance. (**A**) Phylum. (**B**) Class. (**C**) Order. (**D**) Family. (**E**) Genus. (**F**) Species level.

Individual PLA analysis revealed that 66.6% (*n* = 2) of the cases were polymicrobial infectious foci. The first case was female (S178), where 18 different genera and species were identified, with *Gardnerella vaginalis* being the predominant microorganism in the PLA with a relative abundance of 0.51, followed by *Lactobacillus iners* and *Sneathia amnii.* For the second case of polymicrobial PLA, it was a male patient (S180), with 18 different genera and species present. The strict anaerobe *Prevotella timonensis* was the dominant bacterium in the infectious focus with 0.51 relative abundance, followed by various strict anaerobes of other species of the genus *Prevotella*, with an overall relative abundance of this genus of 0.66. A case of monomicrobial PLA was identified from a female patient (S179), where *Lactobacillus iners* was identified as the only microorganism in the infectious focus (1.0 in relative abundance). Finally, homology in taxonomic assignment was observed for each PLA case at all six levels analysed. Figure 6A–F show the results of the individual analysis of the taxonomic composition of the PLAs of the patients treated at HJM.

### 3.6. Analysis of the Core Microbiome in the PLA

**Figure 7:**
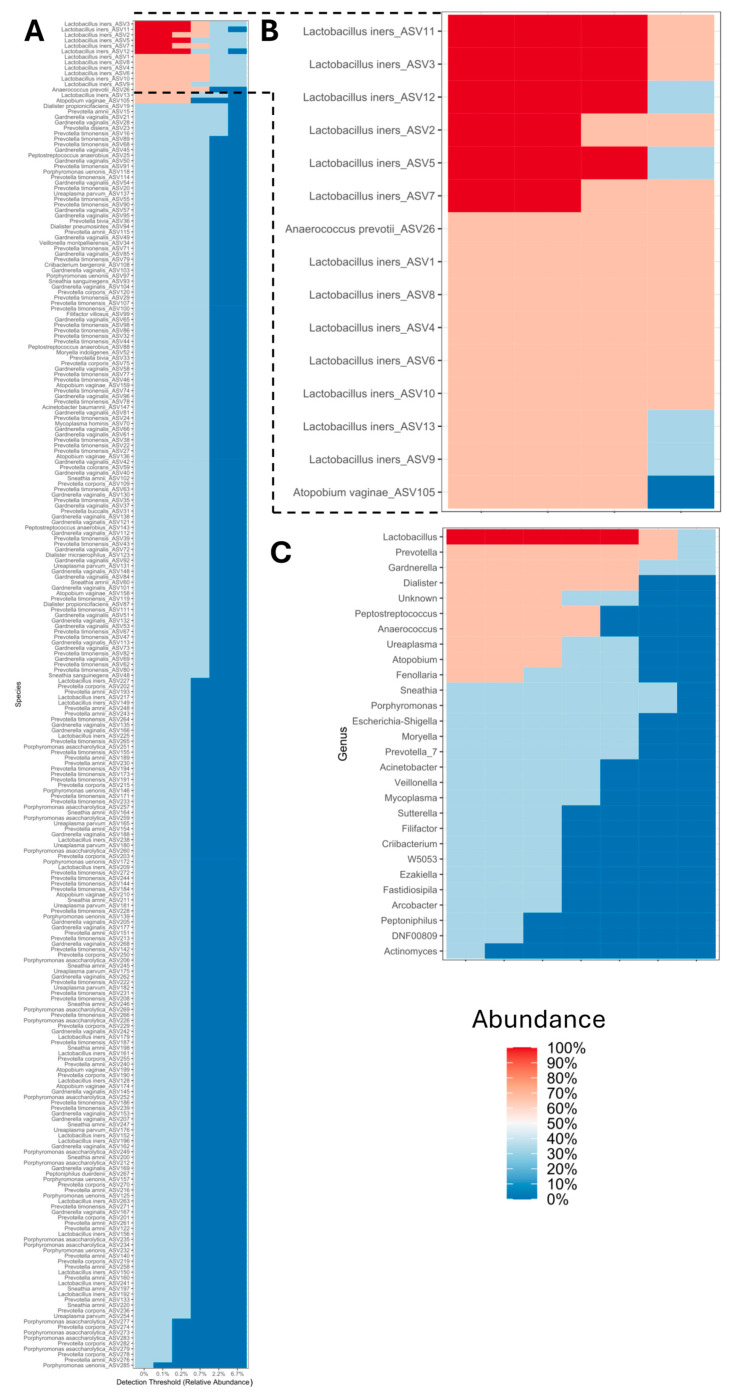
Analysis of the core microbiome of PLAs from patients treated at *Hospital Juárez de México*. (**A**) Global core microbiome, (**B**) genera and species with the highest relative abundance of the core microbiome, and (**C**) genera with the highest relative abundance of the core microbiome.

Core microbiome analysis of the PLA set showed 247 ASVs (Figure 7A), with a predominance of 10 ASVs, represented by bacteria with the highest relative abundances of the genera *Lactobacillus*, *Prevotella*, *Gardnerella*, *Dialister*, unknown group, *Peptostreptococcus*, *Ureaplasma*, *Atopobium*, and *Fenollaria*. Of the genera described above, species represented by *L. iners*, *Anaerococcus prevotii*, and *Atopobium vaginae* were prevalent up to 75% in relative abundance for *L. iners* (Figure 7B,C).

## 4. Discussion

An adequate approach to the antimicrobial treatment of any infectious process depends fundamentally on an accurate and timely diagnosis in the identification of the aetiological agent, and PLA is undoubtedly one of them. Due to the fact that the diversity of bacterial agents that have been reported as causative agents of PLA is limited and only in some cases they form polymicrobial infectious foci, culture methods show weaknesses because they are directed procedures, i.e., they are oriented towards the search for microorganisms that the scientific literature has reported as the most frequent, with enterobacteria being one of them [32,33]. Nevertheless, there are microorganisms that have been omitted, especially in laboratories with few resources, due to their slow growth, nutritional requirements, and particular incubation characteristics, among others. This is why the limitations of traditional culture methods of purulent material from PLA as diagnostic methods are increasingly recognised, even though it has been described as the gold standard. This is because traditional culture methods have been reported to fail to detect causative bacteria in 30–38% of cases or may fail to detect all causative bacteria [18,19].

This weakness is reflected in diagnostic bias as it impacts on the resolution of the infection, and in some cases increased morbidity and mortality due to treatment failures in eradicating the causative agent(s). Therefore, to provide information on causative agents that may go undetected in classical culture, the aim is to show evidence obtained by massive sequencing of the *16S rRNA* gene of cases of PLA with absence of growth in classical microbiological culture. In particular, the identification of bacteria with significant relative abundances and little association with this type of infection, such as *L. iners*, *G. vaginalis*, and *P. timonensis*, shows not only the polymicrobial characteristics of PLA in two of the cases, but also the limitations of traditional culture diagnostic approaches (Figure 5, Figure 6 and Figure 7). The literature has shown that positive results in pus cultures of PLA are represented by Gram-negative aerobic bacilli such as *Enterobacterales* (*E. coli* and *Klebsiella* spp.) and anaerobes such as *Bacteroides*, *Fusobacterium* spp., *Clostridium* spp., *Enterococcus* spp., and *Streptococcus viridans* group [34,35,36,37].

However, polymicrobial infections have now also been recognised, with few studies addressing them due to limited simultaneous identification methods [38]. The identification of 100% relative abundance of *Lactobacillus iners* in a PLA of a female patient (179), a microorganism generally associated with the vaginal microbiota, highlights the ability of microorganisms considered commensal to an anatomically specific area to be pathogenic and, in this case, could generate a PLA. Previous studies have identified bacteria of the genus *Lactobacillus* as causative agents of PLA. For example, the cases of PLA by *Lactobacillus* reported by Omar et al. (2019) and Ramos-Coria et al. (2021), where they indicate that comorbidities such as uncontrolled *diabetes mellitus* is one of the main factors that trigger the emergence of this type of infection [39,40]. Concern about this type of illness due to these bacteria is such that it has been speculated that probiotic consumption could be one of the side effects in susceptible individuals [41]. This finding is particularly relevant, as conventional culture methods failed to detect this microorganism, suggesting that growth requirements may have been an obstacle, as *L. iners* shows optimal growth on MRS agar (de Man, Rogosa and Sharpe), not available in a conventional bacteriology laboratory. Moreover, being an understudied bacterium in settings outside the vaginal microbiota, it might be underestimated and represent an example of how diagnostic approaches are limited. Similarly, the detection of *G. vaginalis* in another female case of PLA (178) suggests an unusual relationship between the vaginal microbiota and PLA. *Gardnerella vaginalis* is a bacterium commonly associated with bacterial vaginosis, and its role in PLA is unusual [42]. The first reported case as a causative agent of PLA was in Alabama, USA, in 1988 by Ezzell and Many [43].

In this case report, it is speculated that the development of pylephlebitis caused by *G. vaginalis* and the possible synergistic interaction of an anaerobic and aerobic organism triggered the development of PLA. Derived from this isolated finding, we conducted an extensive literature review of the available scientific literature on PLA, and to our knowledge, we have identified the second case of PLA due to *G. vaginalis* in a Mexican. patient. However, it is not difficult to speculate that in the context of the difficulty in identifying the causative agents of PLA, *G. vaginalis* has been implicated in undetected cases and is classified as an unknown aetiology in PLA. The fact that this bacterium has been identified by massive sequencing with significant relative abundance and not by culture methods highlights the inherent limitations of classical microbiological culture. Finally, *P. timonensis* is an example of a microorganism of odontogenic origin that is difficult to grow because it is strictly anaerobic and was identified with significant relative abundances, and unlike *L. iners* and *G. vaginalis* has been linked to PLA by culture [44,45,46] and metagenomic tools such as pyrosequencing but remains extremely uncommon to detect [47]. This strict anaerobic bacterium, usually found in the oral microbiome, was identified in this work as the main microorganism (in relative abundance) and could be a causal agent in this case of polymicrobial PLA (S180).

This finding is particularly relevant where culture methods can fail and even more so when dealing with multiple pathogens, especially those requiring anaerobic or microaerophilic culture conditions. Furthermore, in PLA where empirical administration of antimicrobials is common, the ability of culture methods to detect viable bacteria is compromised. The information presented in this work shows the immediate need for the modification of protocols in the microbiological diagnosis of PLA or the implementation of molecular methodologies to provide early diagnosis for timely treatment. The ideal molecular target for such studies is the *16S rRNA* gene, as it is highly conserved among bacteria, allowing for its use in the universal detection of bacterial species. In addition, it contains regions that can be used for genus- and species-specific identification. In particular, the V3-V4 hypervariable region has already been used in our working group in other infectious models and proved to be highly efficient for the resolution of infectious aetiologies negative to classical culture [48]. Although this approach is not routinely used in the diagnosis of infectious diseases (including PLA) and is even somewhat difficult to translate in terms of cost, its implementation could significantly improve the diagnosis and treatment of PLA. Nonetheless, these data should be interpreted with caution as massive sequencing does not provide information on viability and antimicrobial susceptibility. This is why classical microbiological culture remains the gold standard, due to its ability to determine pathogen viability and access to antimicrobial susceptibility testing. However, flaws in the design of culture protocols can lead to significant diagnostic biases. Failure to detect pathogens in PLA may lead to the selection of ineffective antimicrobials, reflecting persistence of infection, and then to the development of relapses, which were not detected in these three cases (Figure 1). The incorporation of comprehensive culture methods in bacteriology laboratories could offer a more robust approach to the diagnosis of infections such as PLA.

## 5. Conclusions

The findings of this study show the need for a critical review of the diagnostic methodologies used in the search for the aetiology of PLA. The identification of unusual microorganisms such as *Lactobacillus iners*, *Gardnerella vaginalis*, and *Prevotella timonensis* by massive sequencing highlights the importance of using molecular tools to characterise complex infections but also highlights the need to improve classical microbiological culture methods to avoid biases that may negatively affect the resolution of the infection and, ultimately, patient survival. Finally, the data shown in this work could generate a bias in the interpretation of the etiological agent of PLA, because they are microorganisms that have been little studied with respect to the microorganisms epidemiologically identified as causal agents of PLA.

## Data Availability

Bello-López, Juan Manuel (2024), “Massive Sequencing of V3-V4 Hypervariable Region in Pyo-genic Liver Abscesses Reveals the Presence of Unusual Bacteria Not Detected by Classical Culture Methods”, Mendeley Data, V1, doi: 10.17632/8gwdjdhbph.1.
